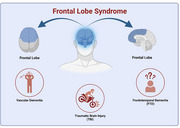# Differentiating Behavioral and Cognitive Symptoms Across Neurodegenerative and Traumatic Etiologies of Frontal Lobe Syndrome

**DOI:** 10.1002/alz70857_098767

**Published:** 2025-12-24

**Authors:** Setareh Rassa, Arman Hajikarim‐Hamedani, Nikan Shafiei Alavijeh, Maryam Noroozian

**Affiliations:** ^1^ Cognitive Neurology, Dementia and Neuropsychiatry Research Center, Tehran University of Medical Sciences, Tehran, Tehran, Iran (Islamic Republic of)

## Abstract

**Background:**

The prefrontal cortex is divided into three main domains: the dorsolateral prefrontal cortex, the medial prefrontal cortex, and the ventral or orbital frontal cortex; functionally, the two latter areas are considered as one uniform structure, the ventromedial prefrontal cortex. Dorsolateral controls cognitive and executive skills, while ventromedial controls emotional processing and behavioral inhibition. Frontal lobe syndrome encompasses clinical disorders caused by prefrontal brain injury and dysfunction. A variety of diseases can induce frontal lobe lesions, including closed head trauma, cerebrovascular disease, tumors, neurodegenerative disease, Multiple Sclerosis, HIV, and others. Frontal lobe syndrome is difficult regardless of cause. Since treatments differ, distinguishing behavioral and cognitive symptoms is vital to treating underlying causes.

**Method:**

Following PRISMA guidelines, studies from PubMed, Scopus, Web of Science, and PsycInfo (1991‐2025) were reviewed. Data were extracted using EndNote 21, focusing on demographics, study characteristics, population details, behavioral symptoms, cognitive symptoms, and neuroimaging. Methodological quality was assessed using modified Cochrane and Effective Public Health Practice Project tools.

**Result:**

A systematic review of clinical and neuroimaging studies reveals that neurodegenerative conditions, such as frontotemporal dementia (FTD), predominantly affect older adults and are characterized by progressive behavioral changes, including disinhibition and apathy, along with cognitive impairments in executive functions. Neuroimaging typically shows frontal and/or anterior temporal atrophy. In contrast, traumatic brain injury (TBI) often impacts a younger demographic, leading to acute behavioral disturbances and cognitive deficits. Neuroimaging findings in TBI vary based on injury severity and location, with damage often observed in the inferior medial frontal lobe.

**Conclusion:**

In conclusion, while FTD and TBI involve frontal lobe dysfunction, they differ in population characteristics, symptom progression, and neuroimaging profiles. FTD presents with gradual behavioral and cognitive decline associated with specific patterns of brain atrophy. In contrast, TBI results in more immediate symptoms with variable neuroimaging findings depending on the nature of the injury. These distinctions are crucial for accurate diagnosis and tailored interventions.